# Reflex syncope due to exaggerated cardioinhibitory vagal response to photic stimulation: a case report

**DOI:** 10.1093/ehjcr/ytaf123

**Published:** 2025-03-07

**Authors:** Rebecca Lewis, Christopher E D Saunderson, Muzahir H Tayebjee, Sam Fairclough, Ben N Mercer

**Affiliations:** Department of Cardiology, Yorkshire Heart Centre, Leeds General Infirmary, Leeds Teaching Hospitals NHS Trust, Great George Street, Leeds LS1 3EX, UK; Department of Cardiology, Yorkshire Heart Centre, Leeds General Infirmary, Leeds Teaching Hospitals NHS Trust, Great George Street, Leeds LS1 3EX, UK; Department of Cardiology, Yorkshire Heart Centre, Leeds General Infirmary, Leeds Teaching Hospitals NHS Trust, Great George Street, Leeds LS1 3EX, UK; Leeds Institute of Cardiovascular and Metabolic Medicine, University of Leeds, 6 Clarendon Way, Leeds LS2 3AA, UK; Adult Neurology, Leeds General Infirmary, Leeds Teaching Hospitals NHS Trust, Great George Street, Leeds LS1 3EX, UK; Department of Cardiology, Yorkshire Heart Centre, Leeds General Infirmary, Leeds Teaching Hospitals NHS Trust, Great George Street, Leeds LS1 3EX, UK

**Keywords:** Reflex syncope, Cardioinhibitory, Vagal, Photic stimulation, Bradycardia, asystole, Trigeminocardiac, Pacemaker, Cardiac pacing, Case report

## Abstract

**Background:**

Transient loss of consciousness is a common presentation to emergency departments, medical assessment units, and cardiology and neurology outpatients. It is a presentation frequently resulting in hospital admission, or multiple presentations to health services and in up to a third of cases a specific aetiology is not identified. [D'Ascenzo F, Biondi-Zoccai G, Reed MJ, Gabayan GZ, Suzuki M, Costantino G, et al. Incidence, etiology and predictors of adverse outcomes in 43,315 patients presenting to the Emergency Department with syncope: an international meta-analysis. *Int J Cardiol* 2013;167:57–62.]

**Case summary:**

A 68-year-old female presented with an unexplained episode of collapse with amnesia. Initial cardiac and neurological investigations were normal. A sleep deprived electroencephalogram (EEG) with intermittent photic stimulation triggered a profound bradycardic response with a 12 second period of asystole interspersed with a single junctional beat. The EEG demonstrated slow delta and theta activity frequency waves without epileptiform spikes or sharp waves during the period of bradycardia consistent with decreased cerebral perfusion. A dual chamber pacemaker was implanted and the patient has remained asymptomatic since implantation.

**Discussion:**

This case highlights an unusually pronounced cardioinhibitory response to photic stimulation successfully treated with pacemaker implantation. ‘Trigeminocardiac’ reflex mechanisms are described which may explain this phenomenon in association with sinus node dysfunction.

**Learning points**

- Photic stimulation may rarely cause a cardioinhibitory response with bradycardia resulting in syncope.
- Identifying mechanism of syncope is pivotal in determining the appropriate management strategy.

## Introduction

Transient loss of consciousness is a common clinical presentation which can offer a significant diagnostic challenge. Here we present a case of exaggerated cardioinhibitory vagal response to photic stimulation associated with likely age-related sinus node dysfunction. Bradycardia related to photic (alternatively described as ‘flash’) stimulation is not a well-defined clinical entity and has only previously been described in case reports.^1^

## Summary figure

**Table:** 

6th June	Patient presents to the Emergency Department with initial episode of collapse.
31st July	Reviewed in Neurology outpatient clinic.
12th August	Initial review in Cardiology outpatient clinic.
4th September	Normal awake electroencephalogram (EEG).
October	Second episode of unresponsiveness.
30th November	Sleep EEG with photic stimulation demonstrating asystolic response.
9th December	Second review in Cardiology outpatient clinic.
17th December	Dual chamber pacemaker implantation.

## Case presentation

A 68-year-old Caucasian female presented to the Accident and Emergency Department with an episode of collapse followed by a few minutes of amnesia. She was subsequently referred on to the Neurology outpatient department for the further assessment. A history from her and her husband revealed she had been standing in the kitchen making a drink, she fell suddenly to the floor, before standing independently and walking to the next room. She could not recall falling or walking between rooms and her next memory was ‘waking up’ on the sofa with her drink. Her husband did not witness her fall but did see her get up and confirmed there was no evidence of seizure activity, abnormal behaviour, or speech disturbance. She subsequently had a further episode of unresponsiveness lasting 20–30 seconds whilst she was a passenger in a car.

She has a history of anxiety and Raynaud’s syndrome. She did not take any regular medications on initial presentation but later commenced Fluoxetine 20 mg once daily. She has never smoked and does not drink any alcohol. She has a family history of ischaemic heart disease but not of epilepsy, cardiomyopathy, or sudden cardiac death.

Physical examination was unremarkable. Lying and standing blood pressure, routine blood tests, electrocardiogram (ECG), computed tomography of the head, and initial electroencephalogram (EEG) were normal. An echocardiogram demonstrated normal biventricular size and function and only mild physiological mitral and tricuspid regurgitation.

**Figure 1:**
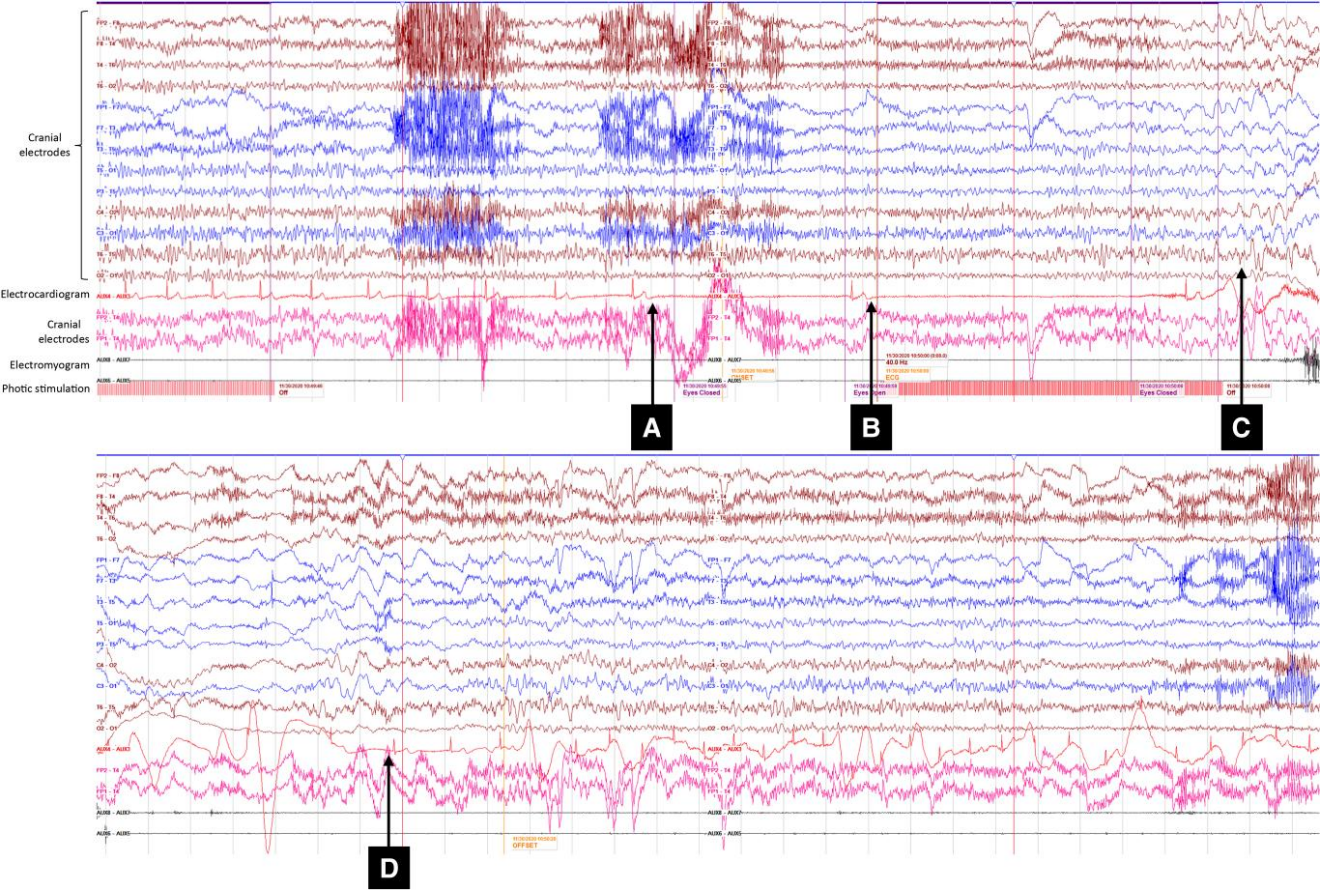
Electroencephalogram recording during 25 hz photic stimulation (*A*) start of 5 s sinus pause (*B*) start of 7.5 s sinus pause (*C*) electroencephalogram demonstrates theta and delta frequencies (*D*) resolution and return of heart rate to baseline.

Following her second episode of unresponsiveness the patient underwent a sleep deprived EEG incorporating intermittent photic stimulation. With photic stimulation at 25 Hz, the heart rate gradually decreased with consecutive 5 and 7.5 second sinus pauses (*Figure 1*). The EEG demonstrated theta and delta activity, consistent with decreased cerebral perfusion. The patient appeared briefly anxious before becoming unresponsive and slumping to her right side. She was immediately laid flat and rapidly regained consciousness as the heart rate increased back to the pre-procedure baseline (66–72 b.p.m.). The EEG rapidly returned to her original background rhythm. The patient had transient retrograde amnesia of the events leading up to the episode, however, within 10 minutes her recollection had returned. She could not recall the event itself. She reported the symptoms experienced during the recording to be identical to the previously described episodes in the community.

As the patient demonstrated profound asystole with limited prodrome we elected to implant a pacemaker. She has remained asymptomatic since with no further syncope in the last 3 years and negligible atrial and ventricular pacing burden (<1%) at serial follow-up.

## Discussion

Vasovagal syncope and epilepsy are distinct entities and may be difficult to distinguish, particularly when syncope is accompanied by anoxic seizures. Furthermore, bradycardia has been well documented during ictal events and has rarely been shown to be the trigger of epileptic seizures.^2–4^

Use of activation procedures, including hyperventilation or photic simulation during EEG may help elicit seizures^5^ and there are case reports of photic simulation leading to bradycardia and seizure like activity.^1^

This case documents a profound bradycardic response following photic stimulation. The EEG demonstrated the evidence of cerebral hypoperfusion as a result of bradycardia rather than any epileptic activity in the form of a photoparoxysmal response.

There are two potential mechanisms to explain this paroxysmal reaction in our patient. The ‘photic sneeze reflex’ (PSR) is also a well described phenomenon in which individuals experience sudden sneezing in response to bright sunlight. Its mechanism is poorly understood. It is suggested that ‘cross-talk’ in the brainstem between optic, trigeminal, and vagus nerve pathways result in the symptoms of nasal altered sensation (trigeminal) and the action of sneezing (vagus).^6^ Other groups suggest a cortical mechanism for the PSR, not something that the EEG in this case would support if this were the mechanism.^7^ Another ‘trigeminocardiac’ reflex, the oculocardiac reflex (Aschner’s reflex), is a known phenomenon whereby pressure to the globe/extraocular muscles results in bradycardia. The proposed pathway is that trigeminal afferents synapse onto vagal nuclei, resulting in a paroxysmal parasympathetic vagal response. Trigeminocardiac reflexes have been widely reported as problematic during maxillofacial surgery as well as skull base surgery.^8^ Significantly, it has been proposed that the PSR may not act via optic nerve afferents, and that in fact the phenomenon is mediated purely by trigeminal afferents detecting temperature change to the face. This would give the PSR a very similar mechanism to the previously described trigeminocardiac reflexes. To our knowledge, however, sneezing on contact with warm air is not a described phenomenon compared with sudden sunlight exposure, which is well described. The PSR is also thought to have a polygenetic basis.^9^

Both reflexes could be considered as ‘trigeminocardiac’. Their evolutionary utility is unclear though they are probably remnants to the ‘diving-reflex’ seen in babies.^10^ One possible explanation in the described case is that photic stimulation led to an alternate vagal response and that instead of sneezing the patient experienced bradycardia in the setting of suspected sinus node dysfunction.

Given the lack of a prodrome, clear temporal correlation with the photic stimulation, prolonged asystolic period and the patient’s age it seems likely that the aetiology of these episodes is a combination of photic-triggered reflex syncope in conjunction with age-related sinus node dysfunction.

As detailed in the ESC Guidelines for diagnosis and management of syncope^11^ identifying a clear cardioinhibitory mechanism was pivotal in determining the appropriate management strategy. In this case pacing was indicated and the risk of recurrent syncope is low. Identifying the underlying pathophysiology was only possible using monitored activation procedures.

## Data Availability

The data underlying this case report are available in the article.

## Lead author biography

Dr Rebecca Lewis, BSc, MBChB is a Cardiology Registrar at Leeds Teaching Hospitals NHS Trust. She is undertaking subspecialist training in Heart Failure and General Internal Medicine. She completed her Bachelor of Science at University of Liverpool in 2007 and MBChB at University of Leeds in 2013.

## Notes

**Consent:** The authors confirm that written consent has been obtained from the patient for the publication of this case in accordance with COPE guidelines.

## Notes

**Funding:** No funding received.

## References

[1] Einspenner M, Brunet DG, Boissé Lomax L, Spiller AE. Bradycardia from flash stimulation. Epileptic Disord 2015;17:409–412. 26575520 10.1684/epd.2015.0775

[2] Monté CP, de Krom MC, Weber WE, de Zwaan C, van Kranen-Mastenbroek VH. The ictal bradycardia syndrome. Acta Neurol Belg 2007;107:22–25. 17569230

[3] van Rijckevorsel K, Saussu F, de Barsy T. Bradycardia, an epileptic ictal manifestation. Seizure 1995;4:237–239. 7582660 10.1016/s1059-1311(05)80067-6

[4] Bergey GK, Krumholz A, Fleming CP. Complex partial seizure provocation by vasovagal syncope: video-EEG and intracranial electrode documentation. Epilepsia 1997;38:118–121. 9024193 10.1111/j.1528-1157.1997.tb01086.x

[5] Verrotti A, Beccaria F, Fiori F, Montagnini A, Capovilla G. Photosensitivity: epidemiology, genetics, clinical manifestations, assessment, and management. Epileptic Disord 2012;14:349–362. 23274161 10.1684/epd.2012.0539

[6] Shetty PA, Bhat S, Jain V, Girish S, Shetty H, Kudlu KP. Implication of photic sneeze reflex in ophthalmology. Indian J Ophthalmol 2023;71:2629. 10.4103/IJO.IJO_107_23 PMC10417945 37322719

[7] Langer N, Beeli G, Jäncke L. When the sun prickles your nose: an EEG study identifying neural bases of photic sneezing. PLoS One 2010;5:e9208. 20169159 10.1371/journal.pone.0009208 PMC2821404

[8] Bhargava D, Thomas S, Chakravorty N, Dutt A. Trigeminocardiac reflex: a reappraisal with relevance to maxillofacial surgery. J Maxillofac Oral Surg 2014;13:373–377. 26224999 10.1007/s12663-013-0541-4 PMC4518787

[9] Wang M, Sun X, Shi Y, Song X, Mi H. A genome-wide association study on photic sneeze reflex in the Chinese population. Sci Rep 2019;9:4993. 30899065 10.1038/s41598-019-41551-0 PMC6428856

[10] Lemaitre F, Chowdhury T, Schaller B. The trigeminocardiac reflex - a comparison with the diving reflex in humans. Arch Med Sci 2015;11:419–426. 25995761 10.5114/aoms.2015.50974 PMC4424259

[11] Brignole M, Moya A, de Lange FJ, Deharo JC, Elliott PM, Fanciulli A, et al 2018 ESC Guidelines for the diagnosis and management of syncope. Eur Heart J 2018;39:1883–1948. 29562304 10.1093/eurheartj/ehy037

